# Exaggerated postural sway improves orthostatic cardiovascular and cerebrovascular control

**DOI:** 10.3389/fcvm.2023.1040036

**Published:** 2023-02-16

**Authors:** Erin L. Williams, Brooke C. D. Hockin, Natalie D. Heeney, Karam Elabd, Helen Chong, Andrew P. Blaber, Stephen N. Robinovitch, Iain T. Parsons, Victoria E. Claydon

**Affiliations:** ^1^Department of Biomedical Physiology and Kinesiology, Simon Fraser University, Burnaby, BC, Canada; ^2^Research and Clinical Innovation, Royal Centre for Defence Medicine, Birmingham, United Kingdom; ^3^School of Cardiovascular Medicine and Sciences, King’s College London, London, United Kingdom

**Keywords:** syncope, postural sway, blood pressure, cardiovascular control, cerebral blood flow, stroke volume

## Abstract

**Introduction:**

Healthy individuals with poor cardiovascular control, but who do not experience syncope (fainting), adopt an innate strategy of increased leg movement in the form of postural sway that is thought to counter orthostatic (gravitational) stress on the cardiovascular system. However, the direct effect of sway on cardiovascular hemodynamics and cerebral perfusion is unknown. If sway produces meaningful cardiovascular responses, it could be exploited clinically to prevent an imminent faint.

**Methods:**

Twenty healthy adults were instrumented with cardiovascular (finger plethysmography, echocardiography, electrocardiogram) and cerebrovascular (transcranial Doppler) monitoring. Following supine rest, participants performed a baseline stand (BL) on a force platform, followed by three trials of exaggerated sway (anterior-posterior, AP; mediolateral, ML; square, SQ) in a randomized order.

**Results:**

All exaggerated postural sway conditions improved systolic arterial pressure (SAP, *p* = 0.001) responses, while blunting orthostatic reductions in stroke volume (SV, *p* < 0.01) and cerebral blood flow (CBFv, *p* < 0.05) compared to BL. Markers of sympathetic activation (power of low-frequency oscillations in SAP, *p* < 0.001) and maximum transvalvular flow velocity (*p* < 0.001) were reduced during exaggerated sway conditions. Responses were dose-dependent, with improvements in SAP (*p* < 0.001), SV (*p* < 0.001) and CBFv (*p* = 0.009) all positively correlated with total sway path length. Coherence between postural movements and SAP (*p* < 0.001), SV (*p* < 0.001) and CBFv (*p* = 0.003) also improved during exaggerated sway.

**Discussion:**

Exaggerated sway improves cardiovascular and cerebrovascular control and may supplement cardiovascular reflex responses to orthostatic stress. This movement provides a simple means to boost orthostatic cardiovascular control for individuals with syncope, or those with occupations that require prolonged motionless standing.

## 1. Introduction

Syncope (fainting; a transient loss of consciousness due to inadequate cerebral perfusion, followed by a spontaneous recovery) (1) is experienced by >35% of individuals over their lifetime (2), and up to 30% of patients with syncope will experience recurrent and severe episodes (3). Syncope can be debilitating, and profoundly impacts patient quality of life (4, 5). Patients with syncope also face an increased risk of fall-related injury (6, 7), with greater morbidity and mortality compared to healthy controls (8).

Directly prior to a faint, patients often experience a variety of prodromal symptoms that make it possible to identify an impending faint (9). Accordingly, presyncopal patients are often advised to perform physical counter-pressure manoeuvres (CPM) (including squatting, muscle tensing, and crouching down with the head between the knees in the “crash” position) to prevent the progression of their symptoms into an episode of frank syncope. These movements promote cardiovascular stability through exploitation of the skeletal muscle pump to promote venous return (1, 10, 11). By increasing muscle pumping in the lower body, syncope can be terminated through improvements in blood pressure, cardiac output (CO), and stroke volume (SV) (10–26), with associated increases in cerebral blood flow velocity (CBFv) (16, 24). Although CPM consistently improve cardiovascular stability in the laboratory setting (11–17, 19–21, 23–34), findings from community based trials indicate that practical limitations to their use can ultimately limit their real-world efficacy (14, 18, 25, 35–37). For example, use of CPM is problematic in patients with a limited prodrome, those with impaired balance or mobility, in situations when adoption of the CPM is not socially acceptable or desirable, or in situations or occupations that require prolonged motionless standing.

Postural sway describes the fine, unconscious movements that occur in upright standing in order to maintain balance. Contractions of the lower limb musculature initiate these movements (38), the magnitude of which depends on several factors. A link between postural sway and skeletal muscle pumping is apparent, as postural movements correspond with body fluid volume shifts (39), and increased postural sway is observed in response to hypotension (40) and cerebral perfusion (41, 42). Accordingly, enhanced postural sway may reflect a subconscious adaptive response to poor orthostatic control (23, 43, 44); it may be that enhanced postural sway could serve as an effective CPM to augment orthostatic cardiovascular control. However, it is unclear whether the small muscle contractions associated with postural sway are sufficient to produce meaningful enhancements in cardiovascular control (45). Given that the direction of postural sway can be manipulated to target specific muscle groups (38, 39), there may be particular sway patterns that optimally bolster cardiovascular control and could be exploited by individuals at risk of syncope. Accordingly, we aimed to examine the effect of various patterns of enhanced sway on cardiovascular and cerebrovascular homeostasis.

## 2. Materials and methods

### 2.1. Participants

This investigation received ethical approval from the Department of Research Ethics at Simon Fraser University (REB#:20190263), and was conducted in accordance with the World Medical Association Declaration of Helsinki (46). A sample size calculation was conducted for the primary outcome measure of systolic blood pressure, based on the magnitude of the anticipated fall in systolic pressure when standing (6), with a difference in means to detect of 5 ± 8 mmHg (the validity of the blood pressure measurement device) (47). These analyses revealed a desired sample size of 23, with a power of 0.8 and alpha of 0.05 (G*Power version 3.1.9.3; Heinrich-Heine-Universität Düsseldorf, Düsseldorf, Germany). We were constrained by the cessation of in person research activities in light of the COVID-19 pandemic, and recruited 20 healthy controls (9 females) to participate in this study before human participant research was terminated. All participants were free of cardiovascular or neurological disease and were not taking cardiovascular acting medication. Participants were excluded if they reported that they experienced recurrent syncope or were pregnant. Testing was timed to avoid menstruation in women, as this may affect OT (48, 49). On the day of testing, participants were asked to have a light breakfast avoiding caffeine, and to avoid strenuous exercise for at least 12 h.

### 2.2. Experimental protocol

**FIGURE 1:**
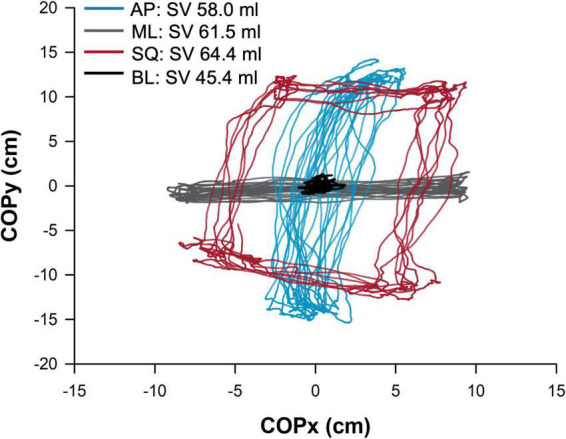
Sample trace showing postural sway in the four conditions in a representative participant. The path (cm) of the centre of pressure is shown, normalized to the origin, during baseline (BL; quiet standing) and exaggerated mediolateral (ML), anterior-posterior (AP), and square (SQ) sway conditions. The participant’s stroke volume (SV) during each condition is provided in the legend. COPx,y, movement of center of pressure in x or y direction.

Physiological testing occurred over a single visit. Prior to testing, all participants provided informed consent, followed by a brief medical history and anthropometric measures of height, weight, and body mass index. Participants were familiarized with the experimental protocol and asked to remove any clothing from their upper body, with the choice of performing testing bare-chested, or wearing a hospital gown. While supine, participants were instrumented with cardiovascular monitoring devices, and initial echocardiographic measurements (inferior vena cava and left ventricular outflow tract diameters) were taken. Participants completed a five-minute supine rest period, followed by a five-minute baseline (BL, quiet standing) postural sway period performed on a force platform. Participants then performed three repetitions of five-minutes of supine rest, and five-minutes of exaggerated postural sway in the anterior-posterior (AP), mediolateral (ML), and square (SQ) directions, which were performed in a randomised order. Testing was terminated upon the participants request, or if the participant experienced symptoms of presyncope, decreased blood pressure (<80 mmHg systolic arterial pressure for greater than five consecutive heart beats), or slowing of the heart rate (HR; new onset bradycardia below 50 bpm). A representative sample trace of postural sway activity during each condition (BL, AP, ML, SQ) is shown in Figure 1.

### 2.3. Cardiovascular monitoring

All cardiorespiratory measurements were recorded continuously throughout testing. The Finometer Pro™ device (Finometer, Finapres Medical Systems, Amsterdam, Netherlands) was used to non-invasively monitor cardiovascular parameters. Recordings were taken at the digital arteries using an inflatable cuff placed on the middle phalynx of the middle finger. Measurements of systolic (SAP), diastolic (DAP), and mean (MAP) arterial pressure were derived using photoplethysmography with reconstruction of the brachial arterial waveform. A lead II electrocardiogram (ECG; Finapres ECG Module, Finapres Medical Systems, Amsterdam, Netherlands) was used to record HR and rhythm. Estimations of beat-to-beat stroke volume (SV) were performed using the ModelFlow algorithm (50). Cardiac output (CO) was calculated as the product of HR and SV. Total peripheral resistance (TPR) was calculated as MAP divided by CO. Doppler ultrasound was used to measure mean CBFv in the middle cerebral artery. CBFv was used as an estimate of cerebral blood flow, as these measures are highly correlated providing the angle of insonation remains constant (51, 52). Accordingly, a 2 MHz ultrasound probe was secured in place over the left temporal window with a headband. A nasal cannula was used to measure end tidal carbon dioxide (P_*ET*_CO_2_) on a breath-by-breath basis (O_2_Cap Oxygen Analyzer, Oxigraf Inc, Sunnyvale, CA, United States).

A pulse-wave Doppler measurement of the left ventricular outflow tract (LVOT) was performed during the last 30 s of each phase of sway by transthoracic echocardiography (CX50, Phillips, USA) by obtaining a modified apical 4-chamber view to open the aortic root (apical 5-chamber view). A further measurement of SV was calculated offline from the product of the velocity-time integral (cm) of the pulsed-wave Doppler in the LVOT and the LVOT cross-sectional area (πr^2^; in cm^2^).

### 2.4. Postural monitoring

Postural sway was measured using a six degree-of-freedom 50 cm × 50 cm force platform (model No. 2535-08, AMTI, Watertown, MA). Raw force platform data were processed using MATLAB software (2020b, MathWorks Inc, Natick, MA, United States). During each standing period, participants stood on the platform without shoes, and with eyes open and their gaze directed forward to a neutral background with no visual cues. Participants were instructed to sway with their body straight, initiating postural sway using the ankle strategy. During exaggerated sway, participants enhanced their sway to the maximum that was comfortable while maintaining a stable foot position, with their heels and toes in contact with the force platform at all time. Center of pressure coordinates were measured and projected onto a two-dimensional plane to allow the calculation of the total path length of postural sway.

### 2.5. Data acquisition

Measurements were sampled at 1 KHz using an analog-to-digital converter (Powerlab 16/30, AD Instruments, Colorado Springs, CO, United States) and stored for offline analysis. Beat-to-beat values of cardiovascular (SAP, DAP, MAP, CO, HR, SV, TPR) and respiratory (P_*ET*_CO_2_) parameters were obtained using LabChart’s peak detection algorithm. R-wave peaks within each QRS complex of the ECG tracing were identified using a threshold voltage. Mean beat-to-beat CBFv was calculated as the area under the curve of each waveform. CBFv data was filtered using a 50 Hz low-pass filter, and median filter (sampling window of 51), prior to analysis. Raw force plate data (COP, total path length, velocity) were low-pass filtered using a 4th order recursive Butterworth filter (5 Hz cut-off frequency).

### 2.6. Time domain analyses

Data were extracted from the final minute of each experimental period (supine rest, standing trials), and used to calculate average values. Cardiovascular measurements were assessed as the absolute change (Δ) between mean supine and standing (sway) values in the final minute of each condition. Outcomes of SV, CO, TPR, CBFv and end-tidal gases were expressed as percentage changes from the corresponding supine value (53). Cardiovascular enhancement of postural sway was measured as the change in the response to the exaggerated postural sway relative to the BL sway condition (where responses were again assessed as the change between mean standing and supine values).

Evaluation of the relationships between postural sway and orthostatic cardiovascular control focused on SAP (because hypotension is a key component of orthostatic syncope), SV (as a proxy for venous return), and CBFv (because the final common pathway for symptoms of orthostatic intolerance is cerebral hypoperfusion).

### 2.7. Frequency domain analyses

Offline beat-to-beat analyses of the digitized ECG, Finometer, and force platform signals were performed at a temporal resolution of 1 ms. Beat-to-beat cardiovascular and postural data were extracted, with postural sway quantified as the total path length of the COP within every R-R interval (RRI). Time series of successive beats were extracted across the entire sway period (5-min) to produce segments approximately 300–500 heartbeats in length (depending on the prevailing heart rate) for SAP, RRI, CBFv, SV, and postural sway (“sway,” total path length in cm). Any significant trends were removed by subtraction of the best polynomial function fitted to the data using low-pass filtering. On three occasions, ectopic beats or artefacts were detected and corrected through linear interpolation of adjacent datapoints, and if present, BL drift was corrected using cubic spline interpolation. To determine heart rate and blood pressure variability, autoregressive monovariate models were fitted to the time series generated from beat-to-beat SAP and RRI (54, 55), and frequency peaks were identified in very low frequency (VLF, < 0.03 Hz), low-frequency (LF, 0.05–0.15 Hz), and high-frequency (HF, 0.15–0.3 Hz) ranges (56). The power and central frequency at each peak were calculated by the computation of residuals (57). We focused our blood pressure analyses on the LF central frequency (LF SAP), as this reflects sympathetic control of the vasculature through the baroreflex (58–60). For heart rate variability we focused on HF RRI peaks, as they reflect parasympathetic regulation of the heart (56).

Cross-spectral analyses were performed between time series data generated from sway with SAP, SV, and CBFv using a bivariate autoregressive model to evaluate their interrelationships. Signal coherence (0-1, 0 indicating no relationship, 1 indicating complete signal alignment) indicates the strength of the relationship between the two variables; values of >0.5 were considered to be statistically significant (61). Discrete measures of phase shift (delay between the sway input signal and cardiovascular output signal, degrees) and transfer function gain (TFG, magnitude of output signal oscillation per unit of input signal) were recorded at the frequency of maximal coherence within the entire frequency spectrum (0–0.5 Hz). Cardiac baroreflex sensitivity (cBRS) was also evaluated using this method to compare the coupling of RRI and SAP during each sway condition, and was quantified as the TFG (ms⋅mmHg^–1^) at the point of maximum signal coherence within the LF frequency band (0.05–0.15 Hz) (62, 63). All phase measurements were converted to time delay (seconds) using the following formula (64):


delay(s)=phase()∘frequency⁢(Hz)-1360

### 2.8. Assessment of cardiopostural coupling

Wavelet transform coherence (WTC) analysis (65–67) was used to identify transient correlations between the postural sway signal and various cardiovascular outcomes (SAP, SV, CBFv), using MATLAB software (2020b, MathWorks Inc, Natick, MA, United States). In this approach, the strength of the relationship between two variables are indicated as a time frequency map, where related aspects of each respective signal can be obtained over specific frequency zones and time points (66).

Assessments focused on the LF frequency (0.05–0.15 Hz), to more closely investigate interrelationships between postural sway and baroreflex-driven sympathetic responses (58–60). Similarly to the spectral method, causal strength between events was defined in terms of coherence (signal interdependence), and response gain (magnitude of relationship) (67). The threshold for significant coherence was determined using the Monte Carlo method (68). The outcome of “fractional time active” estimates the proportion of time that signals were closely aligned relative to the entire sampling period, and was calculated as the area above the significant coherence threshold divided by the area of the entire frequency band (69). “Active path length” was calculated as the product of total path length and fractional time active, quantifying the proportion of the sway path whereby signals of sway and cardiovascular responses were significantly aligned. Similarly, “active gain” was used to estimate the efficiency of the transfer from sway to a given cardiovascular response, quantifying the magnitude of the relationship during periods where the signals were significantly aligned; this outcome was calculated as the product of the response gain and fractional time active (69). To assess the ability of postural sway to directly contribute to a given cardiovascular response, the outcomes of “contribution” (product of response gain and total path length) and “active contribution” (product of contribution and fractional time active) were used.

### 2.9. Statistical analyses

All statistical analyses were performed using Sigmaplot software (Version 14 Systat Software, Inc, San Jose, CA, United States). Data were tested for normality using the Shapiro–Wilk test and are reported as means ± standard error. Statistical significance is reported where *p* < 0.05. Comparisons between outcomes in BL and exaggerated sway (AP, ML, SQ) conditions were made using a one-way repeated measures (RM) ANOVA and a Holm-Sidak *post-hoc* test. Multiple comparisons were accounted for using the Bonferroni correction. Additional comparisons between variables were performed using Pearson’s correlation, and multiple regression analyses.

## 3. Results

### 3.1. Participant demographics

The characteristics of our participants can be found in Table 1. Significant differences in height (*p* = 0.003), weight (*p* = 0.003), and SAP (*p* = 0.03), were detected between male and female participants.

**TABLE 1:** Participant demographics.

	All	Male	Female
*N*	20	11	9
Age (y)	27 ± 2	28 ± 3	25 ± 2
Height (cm)	172.7 ± 2.0	178.0 ± 1.6	166.1 ± 2.9*
Weight (kg)	69.1 ± 2.4	75.4 ± 1.9	61.5 ± 3.3*
BMI (kg⋅m^–2^)	23.1 ± 0.6	23.8 ± 0.5	22.3 ± 1.1
SAP (mmHg)	125.1 ± 1.7	128.4 ± 1.9	120.9 ± 2.5*
DAP (mmHg)	72.4 ± 1.7	73.4 ± 2.1	71.3 ± 3.0
MAP (mmHg)	90.0 ± 1.5	91.7 ± 1.7	87.8 ± 2.5
HR (bpm)	60.8 ± 2.5	56.8 ± 3.1	65.7 ± 3.7

BMI, body mass index; SAP, systolic arterial pressure; DAP, diastolic arterial pressure; MAP, mean arterial pressure; HR, heart rate.

*Significant difference from male participants (*p* < 0.05).

### 3.2. Sway characteristics

Example traces showing postural sway characteristics in a representative participant are shown in Figure 1. Total path length was significantly greater (all *p* < 0.001 vs BL) in all exaggerated postural sway conditions compared to baseline (BL:229.9 ± 15.1 cm, AP:1,986.8 ± 125.6 cm, ML:3,223.2 ± 171.6 cm, SQ:3,147.1 ± 181.2 cm; *p* < 0.001 [main effect]), while the central frequency of sway was significantly higher (all *p* < 0.001 vs BL) during exaggerated sway conditions (BL:0.05 ± 0.01 Hz, AP:0.40 ± 0.02 Hz, ML:0.39 ± 0.02 Hz, SQ:0.34 ± 0.03 Hz; *p* < 0.001 [main effect]). There was also a significant main effect (*p* = 0.031) for the percentage power of sway to be significantly different across conditions (BL:50.3 ± 4.1%, AP:59.0 ± 3.1%, ML:61.7 ± 3.2%, SQ:48.9 ± 2.9%) but post-hoc testing did not reach criteria for statistical significance.

### 3.3. Responses to sway

**FIGURE 2:**
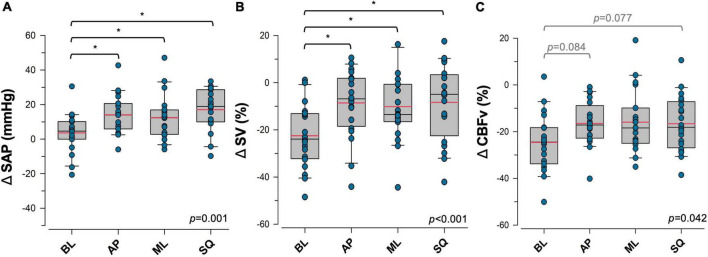
Effects of postural sway on cardiovascular control. Responses to four patterns of sway in **(A)** systolic arterial pressure (SAP), **(B)** stroke volume (SV), and **(C)** cerebral blood flow velocity (CBFv) are shown. Data are presented as the response during each upright postural sway condition, relative to the supine resting values. Significant main effects are indicated in the bottom right corner of each plot. *Significantly different from baseline (*p* < 0.05). Trends that were approaching significance are given in grey. Red = sample mean, black = sample median. BL, baseline (quiet standing) sway; AP, anterior-posterior sway; ML, mediolateral sway; SQ: square sway.

The cardiovascular responses to exaggerated sway are shown in Figure 2. Differences between sway conditions were identified, however there was no apparent effect of sex in these responses. ΔSAP was significantly higher during exaggerated postural sway compared to the BL condition (BL:+3.6 ± 2.5 mmHg, AP:+14.1 ± 2.6 mmHg, ML:+12.4 ± 2.9 mmHg, SQ:+17.1 ± 2.8 mmHg; *p* = 0.001 [main effect]). Significant increases from BL were detected during AP (*p* = 0.017), ML (*p* = 0.038), and SQ (*p* = 0.001) sway. Although ΔDAP was unaffected by exaggerated postural sway, changes in ΔSAP drove a significant improvement in ΔMAP compared to BL (BL:+5.8 ± 1.9 mmHg, AP:+11.4 ± 1.9 mmHg, ML:+9.4 ± 2.0 mmHg, SQ:+12.7 ± 1.8 mmHg; *p* = 0.022 [main effect]). ΔMAP was significantly increased from BL during SQ (*p* = 0.022), but not AP or ML sway patterns.

Exaggerated postural sway did not significantly impact the ΔHR response (BL:+8.5 ± 1.2 bpm, AP:+9.6 ± 1.2 bpm, ML:+8.6 ± 1.4 bpm, SQ:+9.9 ± 1.1 bpm; *p* = NS [main effect]), however, exaggerated sway blunted orthostatic reductions in ΔSV (BL:-22.5 ± 3.0%, AP: –8.6 ± 3.2%, ML: –10.1 ± 3.34%, SQ: –8.3 ± 3.6%; *p* < 0.001 [main effect]), and ΔCO (BL: –10.8 ± 3.7 L min^–1^, AP:+7.7 ± 4.5 L min^–1^, ML:+3.4 ± 3.8 L min^–1^, SQ:+8.6 ± 5.3 L min^–1^; *p* < 0.001 [main effect]). Improvements were detected during AP (ΔSV:*p* < 0.001, ΔCO:*p* < 0.001), ML (ΔSV:*p* = 0.002, ΔCO:*p* = 0.004), and SQ (ΔSV:*p* < 0.01, ΔCO:*p* < 0.001) patterns of sway. Orthostatic reductions in ΔCBFv were also blunted by exaggerated sway (BL: –24.6 ± 2.9%, AP: –16.5 ± 2.1%, ML: –15.9 ± 3.1%, SQ: –16.6 ± 2.7%; *p* = 0.042 [main effect]), with emerging trends of increased ΔCBFv during AP (*p* = 0.077) and SQ (*p* = 0.084) sway patterns. There were no significant changes in ΔP_*ET*_CO_2_ (BL: –4.4 ± 2.6%, AP: –4.9 ± 1.4%, ML: –4.6 ± 1.8%, SQ: –5.4 ± 1.6%; *p* = NS) during exaggerated sway compared to BL. There was a main effect of a greater orthostatic reduction in ΔP_*ET*_CO_2_ in female participants (Males: –3.3 ± 1.3%, Females: –6.7 ± 1.2%, *p* = 0.044), however sex differences within sway conditions were not detected. There were no other sex differences in responses to sway.

Parasympathetically-mediated cardiac responses to sway based on HF RRI were not significantly different between sway conditions (BL:9.1 ± 1.6%, AP:13.4 ± 2.7%, ML: 14.0 ± 1.9%, SQ:13.7 ± 2.6%; *p* = NS).

**FIGURE 3:**
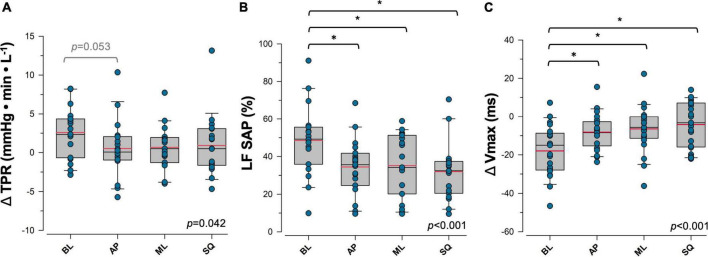
Sympathetic response to postural sway. Responses to four patterns of sway in **(A)** total peripheral resistance (TPR), **(B)** percent power in low frequency systolic arterial pressure variability (LF SAP), and **(C)** maximum transvalvular ejection velocity (Vmax) are shown. Data for panels **(A)** and **(C)** are presented as the response during upright postural sway relative to supine rest. Main effects are indicated in the bottom right corner of each plot. *Significantly different from baseline (*p* < 0.05). Trends that were approaching significance are given in grey. Red = sample mean, black = sample median. BL, baseline (quiet standing) sway; AP, anterior-posterior sway; ML, mediolateral sway; SQ: square sway.

Sympathetically-mediated responses to postural sway are shown in Figure 3. During exaggerated sway, the magnitude of the increase in ΔTPR that occurs with standing was reduced (BL:+2.58 ± 0.07 mmHg⋅min⋅L^–1^, AP:+0.51 ± 0.89 mmHg⋅min⋅L^–1^, ML:+0.67 ± 0.62 mmHg⋅min⋅L^–1^, SQ:+0.91 ± 0.93 mmHg⋅min⋅L^–1^; *p* = 0.042 [main effect]), with ΔTPR during the AP sway pattern approaching significance in comparison to BL (*p* = 0.053). LF SAP was significantly decreased (BL:48.5 ± 4.2%, AP:34.4 ± 3.4%, ML:35.1 ± 3.8%, SQ:31.9 ± 3.4%; *p* < 0.001 [main effect]), and ΔVmax was significantly increased (BL: –18.0 ± 2.9 ms, AP: –8.1 ± 2.0 ms, ML: –6.5 ± 2.7 ms, SQ: –4.2 ± 2.6 ms; *p* < 0.001 [main effect]) during exaggerated sway. Significant differences in these variables were detected during AP (LF SAP:*p* = 0.007, ΔVmax: *p* = 0.003), ML (LF SAP:*p* = 0.016, ΔVmax:*p* < 0.001), and SQ (LF SAP:*p* < 0.001, ΔVmax:*p* < 0.001) patterns of sway compared to BL. cBRS was significantly greater during the SQ sway pattern only (*p* = 0.025) relative to BL (BL:10.9 ± 1.2 ms⋅mmHg^–1^, AP:11.7 ± 1.2 ms⋅mmHg^–1^, ML:11.6 ± 1.3 ms⋅mmHg^–1^, SQ:15.5 ± 1.9 ms⋅mmHg^–1^; *p* = 0.015 [main effect]), and was significantly correlated with ΔVmax responses (*R* = 0.25, *p* = 0.025).

### 3.4. Sway efficacy

**FIGURE 4:**
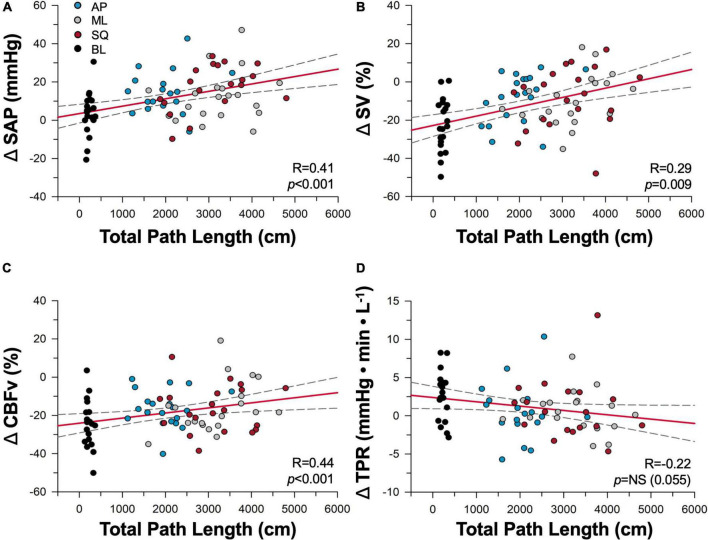
Dose-dependence of cardiovascular responses to sway. Correlations between total path length of sway and cardiovascular responses of **(A)** systolic arterial pressure (SAP), **(B)** stroke volume (SV), **(C)** cerebral blood flow (CBFv), and **(D)** total peripheral resistance (TPR) are shown during baseline (black), anterior-posterior (blue), mediolateral (grey), and square (red) conditions. Regression lines are shown in red, and 95% confidence intervals shown with black dotted lines. NS, not significant.

Relationships between postural sway and cardiovascular responses are shown in Figure 4. ΔSAP, ΔSV and ΔCBFv responses were positively correlated with total path length, whereby greater enhancements in postural sway produced greater improvements in cardiovascular and cerebrovascular control; trends with ΔTPR were emerging (*p* = 0.055) but did not meet criteria for statistical significance. Measures of LF SAP (*R* = –0.43, *p* < 0.001) and ΔVmax (*R* = 0.41, *p* < 0.001) were also correlated with total path length, however there was no relationship between cBRS and total path length.

**FIGURE 5:**
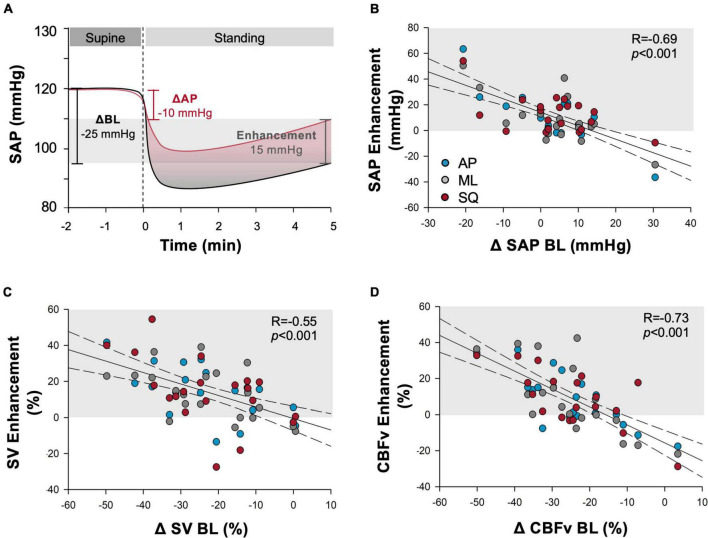
Cardiovascular enhancements from exaggerated sway. **(A)** Example depiction of the “enhancement” outcome measure calculation. Change (upright-supine) in cardiovascular measure during exaggerated sway is subtracted from change during baseline (BL) stand. Outcome quantifies the improvement provided by exaggerated sway in comparison to the deficit experienced during quiet standing. Light grey shading shows magnitude of enhancement experienced. Additional panels report correlations between the enhancement produced by exaggerated postural sway and baseline cardiovascular responses shown across outcomes of **(B)** systolic arterial pressure (SAP), **(C)** stroke volume (SV), **(D)** cerebral blood flow (CBFv) during anterior-posterior (blue), mediolateral (grey) and square (red) patterns of exaggerated sway. Baseline cardiovascular responses were calculated as the change from supine values. Regression lines are shown in black solid lines, and 95% confidence intervals shown with dotted lines. Light grey shading indicates responses that showed cardiovascular benefit during exaggerated sway. Participants experiencing larger decrements in cardiovascular (SAP, SV) and cerebrovascular (CBFv) parameters during prolonged quiet standing in the BL sway condition experienced greater benefit from enhanced postural sway.

Additionally, the SV enhancement during exaggerated sway (calculated as the change from supine during exaggerated sway minus the change from supine during BL sway, Figure 5A) was positively correlated with participant height (*R* = 0.30, *p* = 0.0182); whereby taller individuals had larger increases in SV during exaggerated sway. The cardiovascular enhancement of postural sway was also negatively correlated with the BL sway response in ΔSAP, ΔSV, and ΔCBFv, whereby those with the greatest decrement in cardiovascular parameters during BL sway had the largest benefit with exaggerated sway (Figure 5). The SV enhancement was also correlated with BL total path length (*R* = –0.40, *p* = 0.002) and participant height (*R* = 0.30, *p* = 0.018).

With a multiple linear regression, we assessed the ability of physical parameters (total path length during the exaggerated sway conditions, BL total path length, sway frequency, and participant height) to predict sway efficacy. Variables of BL total path length (β = –0.11, *p* < 0.001) and height (β = 0.62, *p* = 0.004) were effective in predicting the SV enhancement during exaggerated sway (regression *p* < 0.001).

### 3.5. Interdependence of cardiovascular and sway signals

**FIGURE 6:**
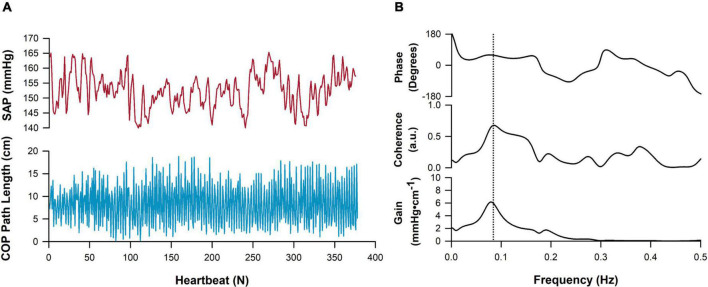
Representative tracing of cross-spectral analysis. Cardio-postural data collected in the mediolateral direction are shown. **(A)** A timeseries of systolic arterial pressure (SAP, red) and postural sway movement, measured as center of pressure (COP) path length (blue) across approximately 350 heartbeats. **(B)** Cross-spectral analysis showcasing the frequency-domain relationship between SAP and postural sway time series. Peak coherence in this participant is denoted by the vertical dashed line and was detected at approximately 0.08 Hz.

**FIGURE 7:**
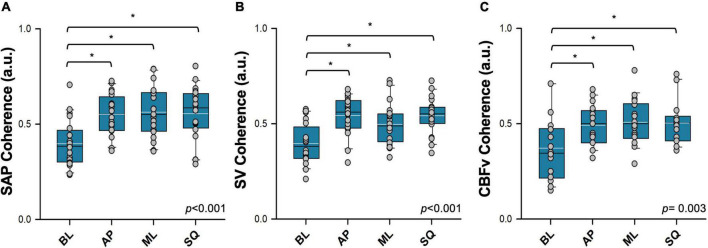
Coherence between postural sway and cardiovascular responses assessed using cross-spectral analysis. Coherence between postural sway and **(A)** systolic arterial pressure (SAP), **(B)** stroke volume (SV), **(C)** cerebral blood flow (CBFv) are shown. Absolute values of mean coherence in each condition are reported. Signals were recorded during quiet standing (BL; baseline sway), and in three exaggerated sway patterns (AP: anterior-posterior sway; ML: mediolateral sway; SQ: square sway). Main effects are indicated in the bottom right corner of each plot. *Significantly different from baseline (*p* < 0.05). Black = sample mean, white = sample median.

A representative example tracing showing cross-spectral analyses alongside raw time series data is shown in Figure 6. Note that coherence thresholds between sway and cardiovascular parameters were not met in all participants, particularly during the BL sway condition (Table 2). In each case, coherence increased during all exaggerated sway conditions (Figure 7). TFG was significantly lower during exaggerated sway (SAP:*p* < 0.001, SV:*p* < 0.001, CBFv:*p* < 0.001).

**TABLE 2:** Cross-spectral analysis of sway and cardiovascular parameters.

	BL	AP	ML	SQ
**Sway – SAP**				
*N*	20	20	20	20
Proportion coherent (%)	20	60	65	75
Coherence (a.u.)	0.40 ± 0.03	0.55 ± 0.02*	0.56 ± 0.03*	0.56 ± 0.03*
Frequency (Hz)	0.159 ± 0.030	0.136 ± 0.016	0.171 ± 0.033	0.136 ± 0.026
TFG (mmHg⋅cm^–1^)	19.1 ± 5.3	5.1 ± 0.9*	3.5 ± 0.7*	2.4 ± 0.4*
Phase (°)	17.7 ± 21.1	31.5 ± 15.8	41.8 ± 11.9	9.2 ± 15.7
Time delay (s)	3.47 ± 2.04	0.10 ± 0.34	2.66 ± 1.27	2.02 ± 1.54
**Sway – SV**				
*N*	20	20	20	20
Proportion coherent (%)	25	65	40	75
Coherence (a.u.)	0.40 ± 0.02	0.54 ± 0.02*	0.50 ± 0.02*	0.54 ± 0.02*
Frequency (Hz)	0.170 ± 0.04	0.190 ± 0.03	0.179 ± 0.027	0.130 ± 0.02
TFG (ml⋅cm^–1^)	15.5 ± 4.2	4.0 ± 1.0*	3.1 ± 0.5*	2.4 ± 0.4*
Phase (°)	9.1 ± 20.9	–43.5 ± 18.3	–53.6 ± 14.2	–29.2 ± 14.1
Time delay (s)	–0.71 ± 1.41	–1.99 ± 1.00	–1.13 ± 0.46	0.25 ± 1.41
**Sway – CBFv**				
*N*	18	19	18	18
Proportion coherent (%)	17	53	50	56
Coherence (a.u.)	0.37 ± 0.04	0.49 ± 0.02*	0.51 ± 0.03*	0.50 ± 0.03*
Frequency (Hz)	0.178 ± 0.04	0.150 ± 0.04	0.173 ± 0.04	0.164 ± 0.03
TFG (cm⋅s^–1^⋅cm^–1^)	12.8 ± 2.8	3.5 ± 0.6*	3.6 ± 1.0*	2.1 ± 0.5*
Phase (°)	–36.9 ± 17.9	13.1 ± 15.5	–1.3 ± 17.2	–22.8 ± 15.7
Time delay (s)	–1.88 ± 1.69	–0.59 ± 0.85	–1.57 ± 1.80	–2.22 ± 1.94

TFG, transfer function gain; SAP, systolic arterial pressure; SV, stroke volume; CBFv, cerebral blood flow velocity; BL, baseline sway; AP, anterior-posterior sway; ML, mediolateral sway; SQ, square sway; a.u., arbitrary units.

*Significant difference from baseline (*p* < 0.05).

The results of the WTC analyses are summarised in Table 3. Coherence between sway and SAP was significantly greater during exaggerated sway (*p* = 0.017), but this was not the case for SV and CBFv. During exaggerated sway, response gain was significantly lower (SAP:*p* < 0.001, SV:*p* < 0.001, CBFv:*p* < 0.001), however the contribution of sway to cardiovascular responses (product of gain and path length) was significantly greater (SAP:*p* < 0.001, SV:*p* < 0.001, CBFv:*p* = 0.001). There were also significant increases in fractional time active (SAP:*p* < 0.001, SV:*p* = 0.013, CBFv:*p* = 0.009) and active contribution (product of contribution and fractional time active; SAP:*p* < 0.001, SV:*p* < 0.001, CBFv:*p* < 0.001) during exaggerated sway.

**TABLE 3:** Wavelet analysis of sway and cardiovascular parameters.

	BL	AP	ML	SQ
**Sway – SAP**				
Active path length (cm)	23.3 ± 3.8	315.4 ± 44.8*^†^	677.8 ± 77.2*	652.9 ± 65.5*^‡^
Coherence (a.u.)	0.71 ± 0.01	0.73 ± 0.01	0.74 ± 0.01*	0.74 ± 0.01*
Response gain (mmHg⋅cm^–1^)	31.3 ± 7.8	8.1 ± 1.1*	6.3 ± 0.7*	5.2 ± 0.6*
Fractional time active (a.u.)	0.10 ± 0.01	0.16 ± 0.02	0.22 ± 0.02*	0.21 ± 0.02*
Contribution (mmHg)	6,025.7 ± 1,218.3	15,109.1 ± 1,674.6*	19,155.1 ± 1,880.1*	15,566.1 ± 1,846.4*
Active contribution (mmHg)	742.4 ± 247.7	2,503.8 ± 481.4*^†^	4,341.9 ± 790.6*	3,307.3 ± 454.6*
**Sway – SV**				
Active path length (cm)	29.5 ± 4.0	289.4 ± 40.4*^†^	632.2 ± 66.3*	521.6 ± 54.9*^‡^
Coherence (a.u.)	0.72 ± 0.01	0.73 ± 0.01	0.74 ± 0.01	0.73 ± 0.01
Response gain (ml⋅cm^–1^)	25.0 ± 6.3	0.78 ± 1.2*	5.0 ± 0.7*	4.5 ± 0.5*
Fractional time active (a.u.)	0.13 ± 0.02	0.14 ± 0.02	0.20 ± 0.02*	0.17 ± 0.02
Contribution (ml)	4,859.5 ± 1,024.1	14,304.8 ± 1,877.6*	15,808.8 ± 1,982.8*	13,612.6 ± 1,488.9*
Active contribution (ml)	650.3 ± 159.6	1,921.1 ± 266.5^†^	3,383.6 ± 647.4*	2,483.2 ± 461.2*
**Sway – CBFv**				
Active path length (cm)	20.3 ± 3.2	182.9 ± 34.7^†^	517.5 ± 67.5*	488.4 ± 85.9*^‡^
Coherence (a.u.)	0.72 ± 0.01	0.72 ± 0.01	0.73 ± 0.01	0.73 ± 0.01
Response gain (cm^2^⋅s^–1^)	21.8 ± 4.3	9.3 ± 2.3*	7.1 ± 1.2*	4.4 ± 0.9*
Fractional time active (a.u.)	0.09 ± 0.02	0.09 ± 0.02^†^	0.16 ± 0.02*	0.15 ± 0.02
Contribution (cm^3^⋅s^–1^)	4,234.1 ± 622.0	16,388.5 ± 3,663.2	23,370.1 ± 4,788.8*	12,312.7 ± 1,740.6
Active contribution (cm^3^⋅s^–1^)	495.9 ± 137.5	1,655.6 ± 474.7	3,548.1 ± 805.4*	1,724.7 ± 329.6

SAP, systolic arterial pressure; SV, stroke volume; CBFv, cerebral blood flow velocity; BL, baseline sway; AP, anterior-posterior sway; ML, mediolateral sway; SQ, square sway; a.u., arbitrary units.

*Significant difference from baseline (*p* < 0.05).

^†^Significant difference from ML (*p* < 0.05).

^‡^Significant difference from AP (*p* < 0.05).

## 4. Discussion

Here we evaluated enhanced postural sway as a novel CPM to aid in the management of syncope. We showed that, in healthy participants, all three patterns of exaggerated postural sway examined (AP, ML, SQ) were effective in producing beneficial orthostatic cardiovascular and cerebrovascular responses in a dose-dependent manner.

### 4.1. Cardiovascular responses

Exaggerated postural sway was successful in augmenting cardiovascular and cerebrovascular responses to standing, with increases in ΔSAP and ΔSV compared to the BL sway condition that likely reflect improved venous return due to recruitment of skeletal muscle pumping in the lower limbs (38). We were concerned that the effect size of any cardiovascular enhancement with exaggerated postural sway would be small given that these discrete manoeuvres require minimal movement; however, the responses we recorded were similar to those documented during clinically recommended CPM. For example, larger movements of the lower limbs with static CPM (e.g., leg crossing, squatting) improve ΔSAP by 10–20 mmHg (11–13, 20, 21, 25, 26, 29), and improve SV by 5–10% (13, 21) and our data elicited comparable improvements in this population of healthy controls. This is presumably because the dynamic nature of the contraction-relaxation movement required to perform exaggerated postural sway facilitates skeletal muscle pumping activity more effectively than isometric movements. The release of compression with dynamic muscle activation allows for a refilling period in the venous beds prior to the next contraction, providing a substrate for venous return and allowing for a significant cardiovascular response to this small and relatively simple movement. By the same mechanism, intermittent calf compression (70, 71), but not sustained calf compression (72) prevents venous pooling and delays the onset of presyncope. Although large muscular contractions may subsequently produce quite pronounced drops in blood pressure (thereby placing participants at a risk of presyncope or syncope), the mild force of contraction required to produce postural sway appears optimal in bolstering blood pressure.

Improvements in ΔDAP were not observed with exaggerated postural sway, which is expected during this dynamic movement or exercise-like intervention (73), with a reduced reliance on TPR responses during the exaggerated sway conditions. We did not detect any impact of enhanced postural sway on orthostatic ΔHR responses, perhaps due to the simultaneous driving forces of a small exercise HR response (11, 73, 74), alongside baroreflex and length-dependant signalling to decrease HR in response to stabilized blood pressure and enhanced ventricular filling (13).

These hemodynamic improvements attenuated orthostatic drops in CBFv, which is of particular interest in the context of orthostatic cardiovascular control as cerebral hypoperfusion is the final common pathway for syncope. Again, the magnitude of the enhancement in CBFv with exaggerated postural sway was similar to previous reports using static CPM (16, 24, 33). Traditional (static) CPM have been most widely considered in recurrent fainting conditions including vasovagal syncope (11, 12, 15, 17, 25, 26) and orthostatic hypotension (14, 16, 20, 21, 23, 27, 33, 37, 75), with some evidence of efficacy in patients with familial dysautonomia (34, 76). In this study, we considered responses in healthy controls that do not experience fainting in daily living, and would be expected to have intact cerebral autoregulatory control. Given their robust cardiovascular and cerebrovascular responses to orthostatic stress, it is possible that these findings are limited by a ceiling effect and thus underestimate the potential for benefit in patient populations. Under orthostatic stress, changes in cerebral perfusion are directly related to symptoms of presyncope, and fatigue, both of which contribute to patient burden (5, 77, 78). Given the improvement in ΔCBFv we showed in healthy controls, there is potential for this strategy to have a significant and meaningful impact on symptom management, and ultimately, quality of life for those affected by recurrent syncope or healthy individuals at a high risk for orthostatic syncope (e.g., occupations that require prolonged standing).

Interestingly, there were no significant differences in responses to exaggerated postural sway between male and female participants, despite women being more prone to syncope than men (79). This may reflect the mechanical nature of the intervention; during dynamic CPM such as postural sway, the cardiovascular benefit is produced by the mechanical pumping action of the rhythmic contraction and relaxation of the musculature (28, 80). This is in contrast to static CPM, where the cardiovascular recovery is largely driven by an increase in sympathetic drive secondary to sustained muscle contraction (1). Given the known differences in sympathetic control of vascular resistance in men and women (81), it may be that responses to static CPM do exhibit sex differences. It is also possible that anthropomorphic differences in height and muscularity could influence sway behaviour or pumping efficacy, and indeed taller individuals had larger responses to postural sway in the present study. This may reflect greater pooling in those with a longer hydrostatic column, and thus greater potential for fluid mobilisation with sway enhancement. Whether lower limb muscularity influences sway efficacy is unclear and should be investigated in future studies.

The improvements we observed were dose-dependent, whereby greater cardiovascular and cerebrovascular benefits were observed in those with greater increases in postural sway, with responses in ΔSAP, ΔSV and ΔCBFv all positively correlated with total path length. When orthostatic cardiovascular and cerebrovascular responses were bolstered by increased postural sway, reliance on baroreflex-mediated sympathetic activation to maintain cardiovascular control was reduced as evidenced by the reductions in cBRS, vascular sympathetic tone (LF SAP), ΔTPR and ΔVmax (a proxy for both length-dependent and independent regulators of ventricular ejection velocity). These findings are compatible with an earlier report that reduced LF SAP was also observed during leg tensing (a maneuver that is commonly recommended by clinicians), and this was accompanied by improvements in CBFv and cerebral oxygenation (24). The improvement in ΔVmax we observed in the face of reduced sympathetic activation is likely driven by the Frank-Starling relationship, reflecting improved venous return and higher end-diastolic volumes, and this is supported by the increases in ΔSV noted with exaggerated postural sway.

Sway efficacy was evaluated with our metric of cardiovascular enhancement, which quantifies improvements during exaggerated sway relative to the participant’s BL stand (exaggerated sway response minus BL response). Participants experiencing larger decrements in cardiovascular (ΔSAP, ΔSV) and cerebrovascular (ΔCBFv) parameters during prolonged quiet standing in the BL condition (and therefore poorer orthostatic control) also subsequently experienced greater benefit from exaggerated postural sway. As noted above, larger SV enhancements with exaggerated postural sway were also more apparent in taller participants (who would experience larger gravitational fluid shifts), and those that swayed more at BL. Presumably, participants with the most impaired orthostatic cardiovascular responses or the largest gravitational fluid shifts experience greater venous pooling, and therefore, had more blood available for mobilisation by the skeletal muscle pumps during exaggerated postural sway to produce a greater cardiovascular and cerebrovascular benefit. The observation that sway efficacy was most pronounced in those with the largest orthostatic cardiovascular decrement lends support to the potential for this intervention to provide benefit in patients with orthostatic tolerance and severe impairments in orthostatic cardiovascular control.

If those with poor orthostatic cardiovascular control experience the most benefit from sway, exercise training may bring further benefit in this population. Exercise training improves orthostatic tolerance in patients with poor orthostatic tolerance, primarily due to plasma volume expansion and improved baroreflex sensitivity (82). Improved cardiovascular stability is apparent following mixed (82, 83), and aerobic (84, 85) training paradigms, although following a purely strength training regimen, the ability to withstand orthostatic stress did not change despite improvements in muscle mass (86). Here, orthostatic stress was imposed with a passive tilt paradigm, and therefore the skeletal muscle pump was not active. It may be that the strength training regimen would have provided benefit during active standing, or CPM. Indeed, during walking, skeletal muscle pumping is known to be more effective in healthy individuals with greater calf circumferences (87). While the role of muscularity in CPM efficacy has not been widely studied, it is reasonable to suggest that improved muscle pumping ability following lower body strength training would provide a further benefit alongside those of aerobic training for those with poor cardiovascular orthostatic control.

In the present study we did not standardise the augmentation in postural sway. We simply asked participants to deliberately increase their sway according to the protocol being employed to the maximum that they felt comfortable without feeling that their balance was perturbed, and without raising the toes or heels off the force platform. This likely means that not all participants were swaying equally during the conditions tested. We do not consider this a limitation to the study, in fact it more accurately recapitulates the real-life application of postural sway, and enabled us to demonstrate the dose-dependent nature of the response, while incorporating manoeuvres that are practical for use during daily activities.

### 4.2. Signal interdependence

Through our cross-spectral autoregressive analyses, we showed that postural sway was more closely coupled to subsequent cardiovascular responses during exaggerated sway, providing clear evidence that this simple maneuver is sufficient to exploit skeletal muscle pumping to contribute to cardiovascular stability during orthostasis. Coherence with SAP, SV, and CBFv was substantially higher during exaggerated sway, both in terms of the average value and the percentage of the sample that reached the coherence threshold. SAP, SV and CBFv were also aligned with exaggerated sway for a longer proportion of the standing period. Interestingly, during each trial, response gain (quantifying the magnitude of the relationship) was significantly lower. Despite the lower gain, we showed that the interdependence between sway and cardiovascular and cerebrovascular responses was enhanced during the exaggerated sway conditions, with both a greater contribution of sway to cardiovascular responses (the cardiovascular responses as a product of path length) and active sway contribution (the contribution over the proportion of the test where cardiovascular and postural signals were closely coupled). Cumulatively, these data show that relationships between postural movements and cardiovascular and cerebrovascular responses are more closely coupled during periods of exaggerated postural sway. Although each movement produces a smaller cardiovascular response, together, the enhanced coupling and larger sway movements culminate in a larger overall cardiovascular benefit.

While the mechanisms underlying the contribution of lower body movements to support cardiovascular control are relatively well understood, we have a great deal to uncover regarding how this relationship manifests more broadly to maintain cardiovascular stability. Static CPM serve as a tool to mitigate symptoms of syncope and presyncope, with conscious muscle contraction creating mechanical (skeletal muscle pumping) and neurological (contraction leading to increased sympathetic drive) responses that ultimately bolster blood pressure. However, postural sway is a stochastic movement that is generally regulated unconsciously in response to neural feedback, ultimately serving to maintain balance in the upright position (88, 89). Enhanced postural sway during quiet standing has been observed in populations with poor orthostatic cardiovascular control, including patients with Alzheimer’s disease (90), and astronauts after space flight (91). Additionally, healthy non-fainting individuals with poor orthostatic tolerance (during passive orthostatic stress) demonstrate enhanced postural sway (both path length and velocity) during quiet standing, and it may be that this protects them from experiencing syncopal episodes during activities of daily living (43, 44). Whether this reflects that these individuals naturally sway more and therefore have reduced reliance on reflex regulation of orthostatic responses, or that their naturally exaggerated sway is a subconsciously learned mechanism to supplement poor cardiovascular reflex control is not clear (43). It has been suggested that, perhaps, a mechanism or reflex is present that recruits skeletal muscle pumping in response to cerebral hypoperfusion or hypotension. Quiet postural sway does increase in response to cerebral hypoperfusion (42), and comparisons between signals of postural movement and fluctuations in SAP have shown that while there are cardiovascular changes in response to sway, there may also be postural movements that occur following changes in blood pressure (68, 92), or baroreflex signalling (93). While there is a clear role for postural activity in cardiovascular regulation, the mechanism and extent of this role is unclear and requires further investigation.

### 4.3. Clinical insights

These data in young healthy controls highlight the potential for exaggerated postural sway to ameliorate orthostatic syncope and presyncope in patients with orthostatic intolerance. Exaggerated postural sway holds great practical potential as it is not only a discrete movement, but it is also relatively easy to perform because it does not drastically challenge mobility or balance. According to current guidelines (1), CPM are a class IIa recommendation for patients with syncope. While community trials show that these CPM can be beneficial (18, 36, 37), practical barriers are reported that limit their effective use in daily living (45). For example, during a squat, one must lower their center of mass, which requires mobility and strength about the hip, knee, and ankle joints. Further, the orthostatic challenge upon resuming the standing position often causes symptoms to reoccur (14–16, 26). Some commonly recommended CPM do allow patients to remain upright, such as leg crossing, however these require a change in the base of support [the area beneath an object or person, encompassed by every point of contact between them and their supporting surface (94)], which may also challenge the balance and strength of the patient, or even increase their falling risk. Conversely, during postural sway, patients can remain in a comfortable standing position with their base of support unchanged. Patients are often taught many CPM so that they can choose which fits their ability, comfort, and social situation, or combine maneuvers to ensure that symptoms fully resolve (15). All three exaggerated sway patterns (AP, ML, SQ) were equally effective in augmenting cardiovascular responses in healthy participants compared to BL, providing flexibility for individuals to adopt their preferred pattern of sway. Further, postural sway could be easily paired with another CPM to prevent the return of symptoms. While these data demonstrate the orthostatic cardiovascular benefits of exaggerated sway in a cohort of relatively young healthy controls, replication of these findings in a larger sample, and with participants with documented orthostatic intolerance (such as VVS or OH), and a wider age span (children, older adults) is necessary to confirm the utility of exaggerated postural sway to defend against orthostatic syncope.

While the cardiovascular effects of sway are similar to other CPM, one benefit over static CPM is that exaggerated or ‘trained’ postural sway can be employed over longer time periods, providing an ongoing prophylactic measure to be deployed in susceptible individuals. This could be utilised in individuals who do not have sufficient prodrome, or are unable to effectively perform CPM. Indeed, one of the most highly reported barriers to employing CPM is the inability to identify an impending faint (45), and this has been noted by patients with and without a history of prodrome prior to their syncopal episodes (14, 18, 25, 35–37). Similarly, there are distinct advantages of the subtlety of postural sway; CPM may not always be socially acceptable or desirable, and can be impractical in certain situations or occupations that require prolonged motionless standing. The robust cardiovascular and cerebrovascular responses to enhanced sway, coupled with the unique qualities of versatility, accessibility, and simplicity offered by enhanced postural sway highlight its potential benefit for use alongside the CPM most often recommended in the clinic.

## 5. Conclusion

These data demonstrate that exaggerated postural sway, regardless of the pattern of sway employed, is effective in producing beneficial orthostatic cardiovascular and cerebrovascular responses in a dose-dependent manner. Implementing exaggerated postural sway during standing may be beneficial for patients with orthostatic intolerance, or for healthy individuals in highly provoking situations for orthostatic intolerance, to manage their symptoms. This simple dynamic manoeuvre provides similar benefits to traditional CPM with the added benefit of improved versatility and accessibility. Future investigations should evaluate the efficacy of exaggerated postural sway in terminating syncope or presyncope in individuals that experience recurrent and troublesome episodes of syncope, both in the laboratory and community settings.

## Data availability statement

The raw data supporting the conclusions of this article will be made available by the authors, upon reasonable request.

## Ethics statement

The studies involving human participants were reviewed and approved by Department of Research Ethics at Simon Fraser University (REB#:20190263). The participants provided their written informed consent to participate in this study.

## Author contributions

ITP and VEC conceived the study and designed the methodology. VEC secured funding and provided project oversight and administration. BCDH, NDH, ELW, ITP, VEC, and HC conducted the experiments. ELW and VEC wrote the manuscript. All authors contributed to data analysis and interpretation, reviewed, edited and provided critical insight to the work, and provided final approval of the manuscript.
